# Nuclear translocation and activation of YAP by hypoxia contributes to the chemoresistance of SN38 in hepatocellular carcinoma cells

**DOI:** 10.18632/oncotarget.6903

**Published:** 2016-01-12

**Authors:** Xiao-Yang Dai, Lin-Han Zhuang, Dan-Dan Wang, Tian-Yi Zhou, Lin-Lin Chang, Ren-Hua Gai, Di-Feng Zhu, Bo Yang, Hong Zhu, Qiao-Jun He

**Affiliations:** ^1^ Zhejiang Province Key Laboratory of Anti-Cancer Drug Research, College of Pharmaceutical Sciences, Zhejiang University, Hangzhou 310058, China; ^2^ Center for Drug Safety Evaluation and Research of Zhejiang University, Hangzhou 310058, China

**Keywords:** hepatocellular carcinoma, SN38, hypoxia, resistance, yes-associated protein (YAP)

## Abstract

Although hypoxia is a prominent feature contributing to the therapeutic resistance of hepatocellular carcinoma cells (HCC) against chemotherapeutic agents, including the Topoisomerase I inhibitor SN38, the underlying mechanism is not fully understood and its understanding remains a major clinical challenge. In the present study, we found that hypoxia-induced nuclear translocation and accumulation of YAP acted as a survival input to promote resistance to SN38 in HCC. The induction of YAP by hypoxia was not mediated by HIF-1α because manipulating the abundance of HIF-1α with CoCl2, exogenous expression, and RNA interference had no effect on the phosphorylation or total levels of YAP. The mevalonate-HMG-CoA reductase (HMGCR) pathway may modulate the YAP activation under hypoxia. Combined YAP inhibition using either siRNA or the HMGCR inhibitor statins together with SN38 treatment produced improved anti-cancer effects in HCC cells. The increased anti-cancer effect of the combined treatment with statins and irinotecan (the prodrug of SN-38) was further validated in a human HepG2 xenograft model of HCC in nude mice. Taken together, our findings identify YAP as a novel mediator of hypoxic-resistance to SN38. These results suggest that the administration of SN28 together with the suppression of YAP using statins is a promising strategy for enhancing the treatment response in HCC patients, particularly in advanced stage HCC cases presenting hypoxic resistance.

## INTRODUCTION

Hepatocellular carcinoma (HCC) is one of the most frequently occurring cancer worldwide. Because most HCC patients are diagnosed at advanced stages, chemotherapeutic treatment is among the most commonly used strategies of intervention. However, the treatment of advanced-stage HCC remains a major challenge, owing partially to the chemoresistance. Recent studies have shown that the frequently occurring hypoxic microenvironment (hypoxia) in HCC plays a key role in the resistance toward chemotherapeutic drugs such as sorafenib [1, 2] and irinotecan [3, 4] used in the treatment of HCC, and contributes to the poor therapeutic outcome. SN38, an active metabolite of irinotecan, has also encountered similar hypoxic resistance in HCC cells. SN38 exerts its anti-cancer activities by inhibiting topoisomerase I, inducing cell cycle arrest and apoptotic cell death. Our previous study found that under hypoxia, HCC cells are highly resistant to a variety of topoisomerase I inhibitors, including SN38 and TPT [4]. However, the underlying mechanisms remain to be elucidated.

It has been considered that the major regulator of cellular adaptation to hypoxia is the hypoxia-inducible factor-1 (HIF-1) [5–8], whose contribution to chemoresistance has been demonstrated in a variety of cancer cell models, particularly in HCC cells [9–11]. However, the recent findings on the HIF-1-independent regulation of tumor angiogenesis [12–14] and chemoresistance [15–17] under hypoxic conditions have challenged this notion and raised the possibility that some other molecules/signaling pathways may also promote the malignancy under hypoxia.

Recent studies have identified the novel transcriptional co-activator, the Yes-Associated Protein (YAP), as an oncoprotein closely correlated with the development of HCC. YAP is an independent prognostic marker of HCC because it is overexpressed in 62% HCC patients [18], and modulates the liver size and liver tumorigenesis [19–23]. As a transcriptional co-factor, the nuclear YAP initiates the transcription by interacting with the DNA-binding transcription factors EA domain family members 1–4 (TEAD1-4) [24–26]. The nuclear localization is among the most important regulatory mechanisms of YAP, and this process is tightly controlled by a kinase cascade, namely, the Hippo pathway, which comprises the mammalian STE20-like 1/2 (MST1/2) and the large tumor suppressor 1/2 (LATS1/2) in mammalian cells [19, 20]. The MST1/2 phosphorylates and activates LATS1/2, which then phosphorylates YAP, causing its cytoplasmic retention and thereby inhibiting the transcription of YAP target genes, such as *CTGF* and *AREG*, involved in the cell proliferation and survival.

The inhibition of YAP by upstream kinases could be relieved by events such as disturbed cell polarity, loss of cell-cell contact, and mechano-transduction [19, 27–29], activating YAP and promoting cancer cell survival, chemoresistance, metastasis, and the other malignant properties. A recent study by Ma *et al.* demonstrated that hypoxia modulates Hippo signaling through SIAH2-mediated degradation of LATS2, leading to the activation of YAP to promote breast cancer cell proliferation and growth [30]. However, the cellular roles and biological function of YAP in hypoxic HCC remain elusive.

In the present study, we found that hypoxia promoted the chemoresistance of human HCC cells toward SN38, as evidenced by the increased IC_50_ values and reduced apoptosis rates. In hypoxic HCC cells and in the hypoxic regions of the human HCC xenografted models, YAP was predominantly localized to the nucleus, which was accompanied by increased mRNA level of the YAP target genes *CTGF* and *AREG*. The small interfering RNA (siRNA)-mediated suppression of YAP expression sensitized the hypoxic HCC cells to the SN38 treatment. Further studies showed that under hypoxia, the nuclear translocation of YAP was not mediated by HIF-1α but relied on the elevated level of HMG-CoA reductase (HMGCR). Accordingly, the inhibition of HMGCR by statins significantly prevented the nuclear translocation and activation of YAP under hypoxia, and led to the enhanced anti-cancer activity of SN38. These findings suggest that the inhibition of YAP by statins may be a promising therapeutic strategy to combat the hypoxic resistance of HCC cells to SN38.

## RESULTS

### HCC cells were resistant to SN38 under hypoxia

**Figure 1:**
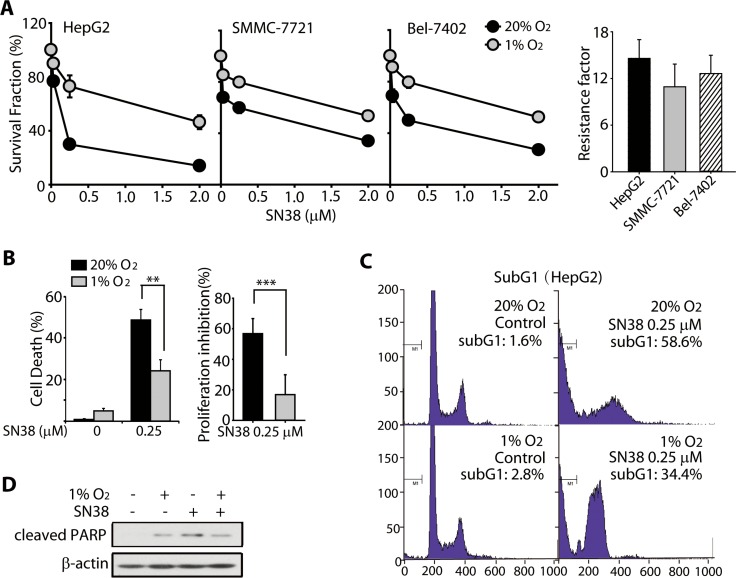
HCC cells were resistant toward SN-38 under hypoxia (**A**) (left) Survival rates of three HCC cell lines, HepG2, SMMC-7721, and Bel-7402, exposed to SN38 for 48 h under normoxic and hypoxic conditions. Cell survival was analyzed using the SRB assay. Black circle: normoixa; gray circle: hypoxia. (right) Hypoxic resistance factors of each cell lines were calculated as IC_50-hypoxia_/IC_50-normoxia_. (**B**) trypan blue exclusion (left) and BrdU proliferation assays (right) were employed to determine the cell death and proliferative inhibition caused by SN38 (0.25 μM, 48 h). (**C**) Sub-G1 population in SN38-treated HepG2 cells under normoxia and hypoxia, as determined by PI staining and FACS analyses. (**D**) HepG2 cells were exposed to SN-38 (0.25 μM) for 48 h under normoxic or hypoxic conditions. The cleavage of PARP was determined by western blot analysis. β-Actin was used as the loading control.

Previous studies have reported the hypoxia-induced resistance of cancer cells to topoisomerase I inhibitors treatment [3, 4]. To confirm this, in the present study, we examined the cell survival and apoptosis in SN38-treated cells under normoxia (20% O_2_) and hypoxia (1% O_2_). The human HCC cell lines HepG2, SMMC-7721, and Bel-7402 were treated with SN38 for 48 h and the cell viability was determined using the SRB assay. As shown in Figure 1A, the survival ratio of SN38-treated cells under normoxia was lower than that of the cells under hypoxia. Specifically, 0.25 μM SN38 significantly decreased the viability of normoxic HepG2, SMMC-7721, and Bel-7402 cells, resulting in the survival rate of 29.8%, 49.8%, 59.7%, respectively, whereas under hypoxia, 0.25 μM SN38 moderately inhibited cell proliferation, as evidenced by the survival rates of 73.0%, 79.5%, and 79.7%, respectively. And the hypoxic resistance factors (IC_50_-hypoxia/ IC_50_-normoxia) were calculated to better illustrate the differential activity of SN38 under hypoxia and normoxia. The resistance factor in HepG2, Bel-7402 and SMMC-7721 cells were 14.58 ± 2.42, 12.57 ± 2.36, 10.93 ± 2.92, respectively (Figure 1A). Additionally, BrdU proliferation [38] and trypan blue exclusion assays [39] were introduced to further display the hypoxia resistance. As shown in Figure 1B (right panel), a significant reduction of proliferation inhibition, determined by BrdU colorimetric assay, was observed in hypoxic SN38-treated groups. The proliferation inhibition ratio dropped from 56.8% (hypoxia) to 16.9% (normoxia). Similar results were obtained from trypan blue exclusion assay (Figure 1B, left panel). SN38 exerted potent cell killing effects on normoxic HepG2 cells (48.8%), whereas under hypoxia, the cell death ratio was significantly reduced (24.1%).

In addition, 0.25 μM SN38 markedly induced apoptosis of normoxic HepG2 cells, with a 58.6% sub-G1 population; but the extent of apoptotic cell death was reduced to 34.4% in hypoxic HepG2 cells (Figure 1C), which was accompanied by reduced cleavage of PARP (MW: 89 kD) (Figure 1D). These data demonstrate that hypoxia confers resistance to SN38 and apoptosis in HCC cells.

### Hypoxia triggered nuclear translocation and total protein accumulation of YAP

**Figure 2:**
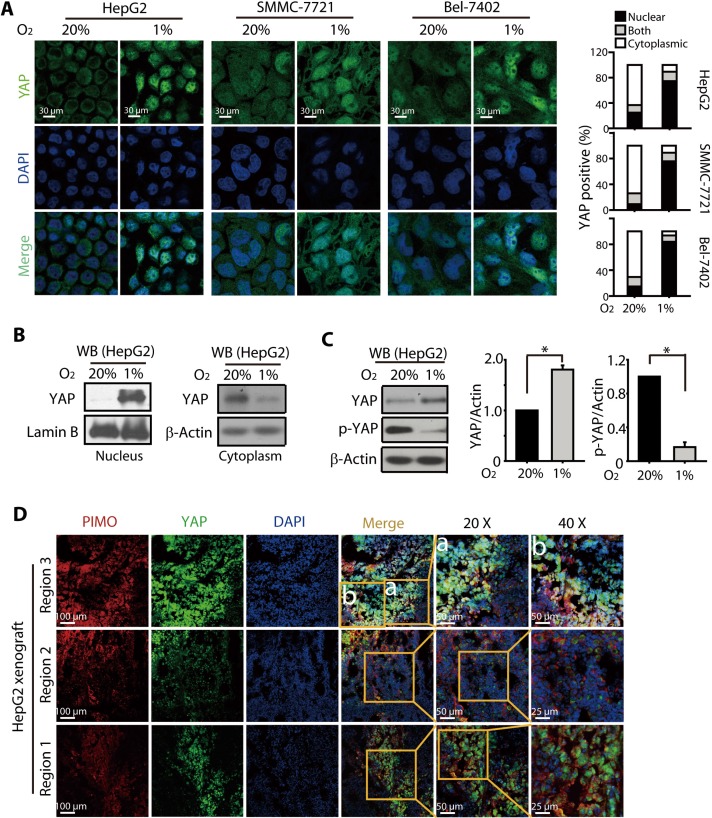
Hypoxia induced YAP nuclear translocation and accumulation in HCC models (**A**) Under hypoxic conditions (24 h), YAP was localized to the nucleus of HepG2, SMMC-7721, and Bel-7402 cells, as determined by immunofluorescence assay. (**B**) Western blot analyses of the nuclear and cytoplasmic fraction of HepG2 cells revealed increased nuclear accumulation of YAP under hypoxia (24 h), when compared to that under normoxia. (**C**) The total protein level of YAP was increased under hypoxia (24 h), which was accompanied by a significant reduction in p-YAP levels. (**D**) YAP was preferentially localized to the hypoxic region (as determined by PIMO, red) in the HepG2-xenografted tumor in nude mice models.

It has been recently reported that hypoxia induces YAP activation by regulating the Hippo signaling pathway in breast cancer cells [30]. However, the precise role(s) of YAP activation under hypoxia remained unknown. Because it appeared that HIFs may not be the sole mediators of hypoxic resistance, we examined whether YAP was activated in the hypoxic HCC cells and contributed to the resistance toward SN38. The results of immunostaining analyses showed that hypoxia triggered a significant nuclear translocation of YAP in HepG2, Bel-7402, and SMMC-7721 cells (Figure 2A). Consistent with these observations, the cell fractionation analysis showed the accumulation of YAP protein in the nuclear fraction of HepG2 cells under hypoxia, whereas little YAP was detected in the nuclear fraction under normoxia. In the contrast, much less cytoplasmic YAP was detected under hypoxia (Figure 2B).

The nuclear translocation of YAP was primarily regulated by phosphorylation. Dephosphorylated YAP translocates into the nucleus, where it binds transcription factors, activating target genes expression while preventing its own cytoplasmic sequestration and subsequent degradation. Therefore to further understand the regulation of YAP by hypoxia, we examined the effect of hypoxia on the phosphorylated and total YAP levels. As shown in Figure 2C, in HepG2 cells, YAP protein level was found to be increased under hypoxia, which was accompanied by a dramatic reduction in the phosphorylated YAP levels. Similarly, the other HCC cells, Bel-7402 and SMMC-7721 also exhibited remarkable loss of p-YAP (Figure S1). To further determine whether a similar trend in the nuclear accumulation and total expression of YAP under hypoxic conditions occurred *in vivo*, xenograft models were created by subcutaneous injection of HepG2 cell suspensions into nude mice, and the hypoxic region was stained with Hypoxyprobe^™^-1 (Pimonidazole Hydrochloride, PIMO). As shown in Figure 2D, the YAP accumulation and its nuclear localization were evident in the PIMO-positive hypoxic regions. In contrast, the PIMO-negative regions showed much less density of YAP.

Taken together, these results indicated that hypoxia caused the nuclear translocation of YAP, likely preventing the phosphorylation and subsequent degradation and leading to the accumulation of YAP.

### Hypoxia-induced YAP activation contributes to the SN38 resistance of HCC cells

**Figure 3:**
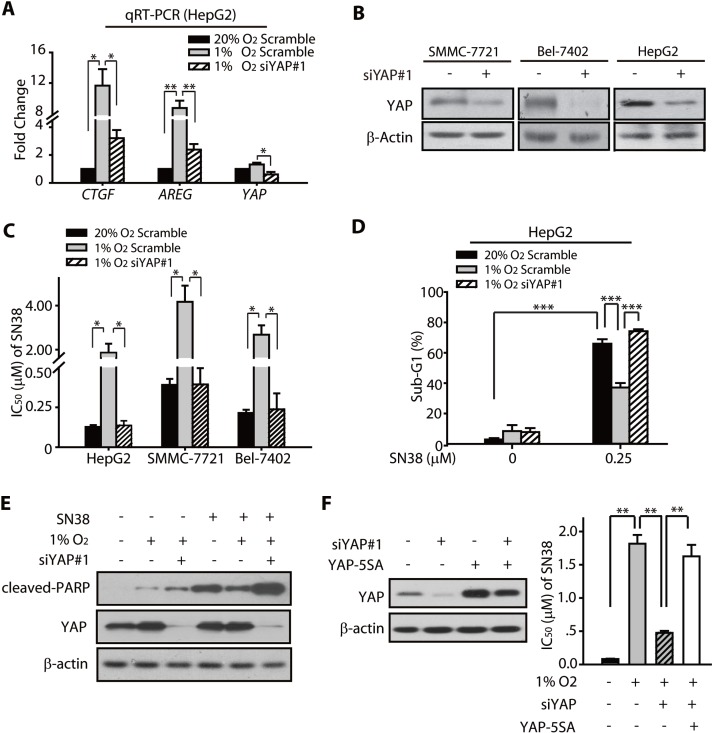
YAP promoted the hypoxic resistance of HCC cells to SN38 (**A**) The qRT-PCR analyses revealed that the mRNA levels of *CTGF* and *AREG* were upregulated under hypoxia (24 h), whereas the YAP siRNA attenuated the increased expression under hypoxia. (**B**) The YAP knockdown was achieved by the transfection of the cells with YAP-targeting siRNA sequence. The SMMC-7721, Bel-7402, and HepG2 cells were either transfected with the negative control (NC) or the YAP-targeting siRNA. (**C**) The knockdown of YAP enhanced the SN38-induced cytotoxicity (48 h) in HCC cells under hypoxia. (**D**) The apoptosis (sub-G1 population) caused by SN38 (48 h) in hypoxic HepG2 cells were enhanced by the YAP knockdown. (**E**) The YAP silencing using siRNA under hypoxia resulted in increased PARP cleavage (48 h) in SN38-treated HepG2 cells. (**F**) Exogenous YAP 5SA was tranfected into YAP depleted HepG2 cells, and attenuated the sensitization of siYAP on the cytotoxicity of SN38 (48 h) under hypoxia.

We analyzed the mRNA levels of the YAP target genes *CTGF* and *AREG*. The results showed that these two genes were significantly upregulated in hypoxic HCC cells (Figure 3A), whereas the knockdown of YAP by siRNA (Figure 3A and 3B) attenuated the increase in the mRNA expression of *CTGF* and *AREG* (Figure 3A). These data suggested that hypoxia-induced nuclear accumulation of YAP led to the activation of its downstream target genes and likely contributed to the hypoxia response, including the chemoresistance.

Therefore, we examined whether the activation of YAP and its target genes under hypoxia contributed to the SN38 resistance. The half maximal inhibitory concentration (IC_50_) values of SN38 in HepG2, Bel-7402, and SMMC-7721 cells transfected with scramble control or YAP siRNA were determined under normoxic and hypoxic conditions. SN38 exhibited much less activity in hypoxic HCC cells than in normoxic cells. Notably, as assessed from the lower IC_50_ values, the YAP knockdown significantly sensitized the hypoxic cells toward SN38 (Figure 3B, 3C, S2A and S2B). Interestingly, hypoxic HCC cells were preferentially sensitized by YAP depletion, as indicated by decreased cell survival (Figure S2C) and remarkable lower hypoxic resistance factors compared to scramble groups (Figure S2D). On the contrary, those normoxic cells were minimally impacted.

Next, we employed PI stating and FACS analyses to evaluate the effect of YAP knockdown in the apoptosis of SN38-treated HepG2 cells. As shown in Figure 3D, the sub-G1 population in the hypoxic SN38-treated cells was significantly less than that under normoxia. However, the YAP depletion effectively rendered the hypoxic cells susceptible to SN38-induced apoptosis. Consistent with this observation, we also found that the cleavage of PARP, which marked the apoptosis, was increased in YAP knockdown SN38-treated hypoxic HepG2 cells compared with their control siRNA-expressing counterparts (Figure 3E).

To further confirm the critical roles that YAP played in the hypoxic resistance to SN38, we introduced YAP (5SA) mutant which lacked five serine phosphorylation sites, insensitive to phosphorylation, thus predominantly located in the nucleus [27]. As shown in Figure 3F, the exogenous mutant of YAP (5SA) significantly rescued the loss of viability of SN38-exposed cells under hypoxia. The IC_50_ values of SN38 under hypoxic were: 1.82 μM for NC siRNA group, 0.48 μM for YAP siRNA group, and 1.63 μM for YAP siRNA + YAP (5SA) group, respectively.

These data collectively indicated the crucial roles of hypoxia-activated YAP in the hypoxic chemoresistance of HCC cells toward SN38.

### YAP activation under hypoxia is HIF-1α-independent

**Figure 4:**
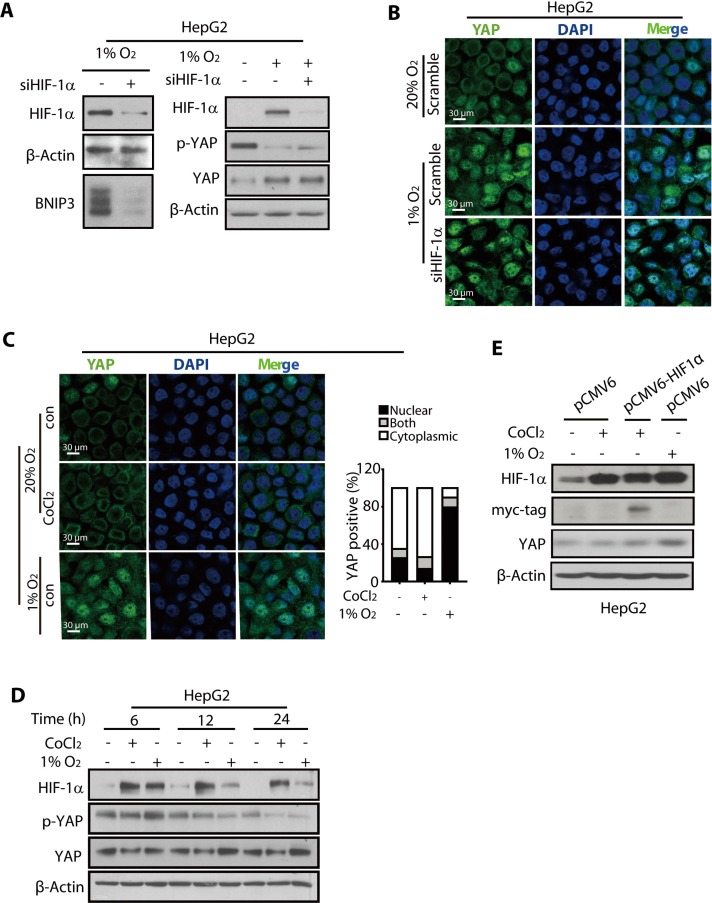
The nuclear translocation and accumulation of YAP was not mediated by HIF-1α (**A**) The HIF-1a knockdown using siRNA had little effect on the reduction in p-YAP levels and increase in the total YAP protein level in hypoxic HepG2 cells. (**B**) The HIF-1α depletion failed to abolish the nuclear localization of YAP under hypoxia (24 h) in HepG2 cells. (**C**) The HepG2 cells treated with CoCl_2_ (200 μM, 24 h) failed to promote nuclear accumulation of YAP in normoxic HepG2 cells. (**D** and **E**) The total YAP protein level of HepG2 cells under normoxia was not increased upon either CoCl_2_ treatment (D) or exogenous expression of HIF-1α (E).

Because HIF-1α has been considered a master regulator of cellular response to hypoxia, we examined whether the hypoxia-mediated activation of YAP was HIF-1α-dependent. For this purpose, we suppressed the expression of HIF-1α using siRNA and used BNIP3, a product of HIF-1α target gene, to monitor the function of HIF-1α (Figure 4A). Immunoblotting and immunofluorescence analyses showed that the reduction in YAP phosphorylation, the increased YAP protein expression, and the nuclear translocation of YAP under hypoxic conditions were not impaired by the depletion of HIF-1α (Figure 4A, 4B and S5A).

Cobalt dichloride (CoCl_2_) has been used to stabilize HIF-1α in cells to mimic hypoxia [40]. Therefore, the HepG2 cells were treated with CoCl_2_ (150 μM) to induce HIF-1α accumulation under normoxia (Figure 4D). However, our results showed that the HIF-1α thus failed to trigger the nuclear translocation of YAP under normoxia, suggesting that the HIF-1α protein under normoxia could not mimic the hypoxia micro-environment, which facilitates the nuclear translocation of YAP (Figure 4C). Additionally, the total YAP level was not affected by CoCl_2_ (Figure 4D), although p-YAP was reduced, raising the possibility that YAP was subjected to some other post-translational modification, such as ubiquitination, caused by CoCl2 [41], thus decreasing the stability.

To further verify that YAP accumulation by hypoxia was not mediated by HIF-1α, we overexpressed HIF-1α by introducing the pCMV6-HIF-1α plasmid into the HepG2 cells. The results showed that HIF-1α overexpression under normoxia, as assessed by the expression level of the Myc-tag, had little effect on the YAP accumulation (Figure 4E). Thus, under normoxia, neither the CoCl_2_-stimulated accumulation of HIF-1α nor its exogenous expression resulted in YAP activation.

Collectively, these results clearly suggested that the hypoxia-induced nuclear translocation and accumulation of YAP was HIF-1α-independent and therefore may be due to some other regulatory mechanism(s), particularly those that are responsive to hypoxia.

### HMGCR-mevalonate pathway mediated hypoxia-induced activation of YAP

**Figure 5:**
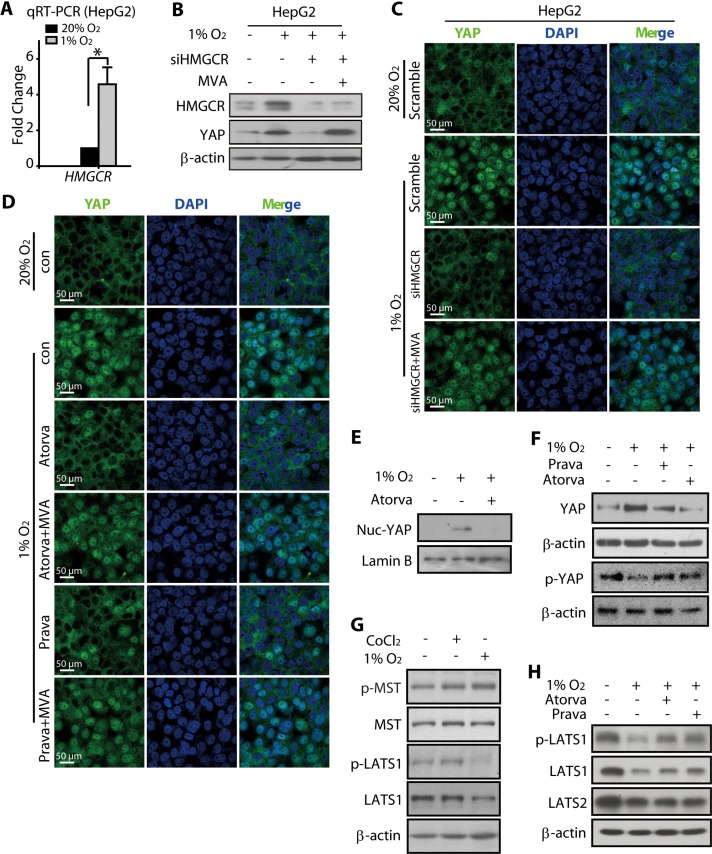
HMGCR-mevalonate pathway contributed to the hypoxia-modulated YAP pathway (**A**) The qRT-PCR analysis showed that the mRNA level of *HMGCR* was upregulated under hypoxia (24 h). (**B**) Hypoxia (24 h) induced the total protein level of HMGCR in HepG2 cells. The knockdown of HMGCR attenuated the YAP accumulation, which could be rescued by treatment with mevalonate (MVA, 5 mM). (**C**) The immunofluorescence analyses showed that the nuclear accumulation of YAP under hypoxia was reduced when HMGCR was depleted, whereas this effect was abrogated by MVA treatment. (**D**) The hypoxia-induced YAP nuclear translocation was prevented by the HMGCR inhibitors atorvastain and pravastain, and the pretreatment of MVA reversed these effects caused by statins. (**E**) Atorvastain exposure for 24 h caused reduced the amount of YAP in the nuclear fraction of hypoxic HepG2 cells. (**F**) Both atorvastatin and pravastain (24 h) reduced the total YAP protein level in HCC cells. (**G**) Hypoxia (24 h) decreased the level of phosphorylated LATS1, which was unaffected in CoCl_2_-exposed cells. (H) Atorvastain and Pravastatin attenuated the hypoxia-induced reduction in p-LATS1 levels in HepG2 cells.

Accumulating evidence suggests the role of cellular metabolism in diverse physiological and pathological processes [42–44]. We were particularly interested in the HMGCR-mevalonate pathway because earlier reports have indicated that this pathway might be upregulated under hypoxia [45]. Therefore, we compared the mRNA and protein levels of HMGCR under normoxic and hypoxic conditions and found that both were upregulated in hypoxic HepG2 cells (Figure 5A and 5B). Notably, the HMGCR knockdown significantly attenuated the nuclear translocation of YAP under hypoxia (Figure 5C and S5B), accompanied with hypoxic accumulation of YAP total protein (Figure 5B). Interestingly, the mevalonic acid (MVA) (0.5 mM, 24 h) treatment greatly reduced the nuclear accumulation of YAP under hypoxia even in the HMGCR knockdown cells (Figure 5C and S5B). Additionally, compared with the HMGCR knockdown cells, the total YAP protein level was also increased in MVA-treated HMGCR knockdown cells (Figure 5B). We then introduced statins, a class of HMGCR inhibitors widely used in the treatment of hypercholesterolemia, to block the MVA production. As shown in Figure 5D and 5E, the treatment with either atorvastatin or pravastatin prevented the nuclear accumulation of YAP under hypoxia, whereas, co-treatment with MVA abolished these effects (Figure 5D and S5C). These results further suggested the involvement of the MVA pathway in the nuclear translocation of YAP under hypoxia. Moreover, the increased accumulation of total YAP protein under hypoxia was also prevented by the treatment with atorvastatin or pravastatin (Figure 5F).

Because it has been reported that the cellular YAP localization and protein stabilization are negatively regulated by two components of the Hippo signaling pathway, namely the LATS and MST kinases, we examined the phosphorylation levels of these two kinases, which reflects their activity. As shown in Figure 5G, neither CoCl_2_ nor hypoxia caused any alterations in protein expression or phosphorylation of the MST kinase. However, the phosphorylated LATS1 (p-LATS1) was significantly reduced in hypoxic HepG2 cells, with a slight reduction in the total protein level also visible under these conditions (Figure 5G). Consistent with this observation, CoCl_2_ treatment failed to produce any effect on the p-LAST1 and total LAST1 (t-LATS1) levels. Interestingly, the hypoxia-induced changes in t-LATS1 and p-LAST1 levels were rescued, at least partially, in HepG2 cells treated with atorvastatin or pravastatin (Figure 5H). These results indicated that the HMGCR-mevalonate pathway played indispensable roles in the activation of YAP under hypoxia, likely through the regulation of LATS1 kinase. The results also suggest that the introduction of HMGCR inhibitors, statins, might be a useful strategy to combat the hypoxia-induced resistance of HCC to SN38.

### Statins sensitized hypoxic HCC cells toward SN38 treatment

**Figure 6:**
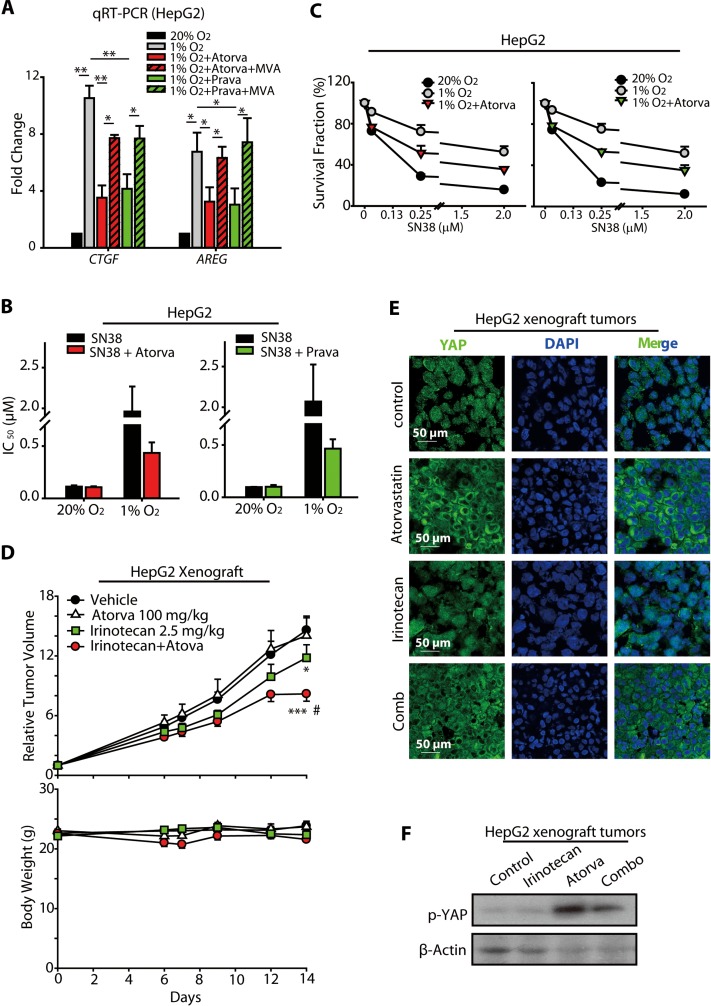
Statins increased the anti-cancer activity of SN38 in hypoxic HCC models (**A**) The induction of *CTGF* and *AREG* mRNA expression was prevented by atorvastain and pravastain treatment under hypoxia. (**B** and **C**) The HepG2 cells were treated with statins and SN38 under normoxic and hypoxic conditions, and the inhibition of cell proliferation was assessed using the SRB assay. The IC_50_ values (B) as well as the survival curves are presented. (**D**) The sensitizing effect of atorvastain on the *in vivo* anti-cancer activities of irinotecan were measured in HepG2 human xenograft models. Nude mice bearing the HepG2 tumors were administered with 2.5 mg/kg irinotecan by i.p. injection every two days and/or 100 mg/kg atorvastain daily by i.p. injection for 14 days. The relative tumor volumes (RTV) are expressed as the mean ± SEM. ***P < 0.001 combination *vs.* vehicle-treated controls; *P < 0.05 irinotecan *vs.* vehicle-treated controls. Body weights are expressed as the mean ± SEM; ^#^*P* < 0.05 combination *vs.* irinotecan. Body weights are expressed as the mean ± SEM. (**E**) The immunofluorescence analyses of HepG2 xenografted tumors treated with atorvastain, SN38 and the combination indicated that atorvastain prevented the nuclear accumulation of YAP. (**F**) p-YAP was increased by atrovastin or the combination of atorvastin and irinotecan.

Because our results showed that statins prevented the nuclear translocation of YAP under hypoxia, we analyzed the effects of statins on the expression of the YAP target genes *AREG* and *CTGF*. As shown in Figure 6A, the increased expression of *AREG* and *CTGF* mRNAs under hypoxia were markedly attenuated by atorvastatin or pravastatin treatment. Additionally, these effects of statins were blocked by the co-treatment with MVA. Together with our observation that YAP is critical for the hypoxia-mediated SN38 resistance in HCC cells (Figure 3), these results not only support our hypothesis that statins could interrupt the hypoxia-induced activation of YAP but also open the opportunity to utilize statins as a potential strategy to combat the SN38 resistance under hypoxia.

To test our hypothesis, we treated the HepG2 cells with or without statins, and then incubated these cells under normoxic or hypoxic conditions. These cells were then subjected to SRB analyses to investigate whether the statins rescued the cells from the hypoxia-mediated SN38 resistance. As shown in Figure 6B, the sensitivity of the cells to SN38 was lower under hypoxia than under normoxia. However, the co-treatment with either atorvastatin (5 μM, 48 h) or pravastatin (10 μM, 48 h) increased the sensitivity of hypoxic HepG2 cell to SN38, although statins alone had little effect on the cell survival. The improved anti-cancer activity of SN38 in the presence of statins was also reflected in its IC_50_ values (Figure 6B). As shown in Figure 6C, co-treatment with atorvastatin enhanced the anti-proliferative effect of SN38 in hypoxic HepG2 cells, with the cell survival fraction declining from 91.40%, 72.29%, and 52.51% to 76.72%, 51.44%, and 35.66%, respectively, in the presence of 0.08, 0.25, and 2.00 μM-SN38, respectively. Similarly, at these concentrations of SN38, the co-treatment with pravastatin reduced the cell survival fraction from 93.48%, 75.05%, and 51.67% to 78.37%, 53.14%, and 34.99%, respectively. These data suggest that the administration of statins along with SN38 may serve as a promising new strategy for overcoming the hypoxia-induced resistance of HCC cells to SN38 treatment.

To further characterize the effects *in vivo*, the combined anti-cancer activity of statins and irinotecan, a prodrug of SN38, was evaluated in HepG2-xenografted models of nude mice. As shown in Figure 6D, the intraperitoneal administration of 2.5 mg/kg irinotecan 3 times per week moderately reduced tumor growth, with a T/C value of 0.81 (mean RTV: irinotecan *vs.* vehicle: 11.81 *vs.* 14.58; *p* = 0.045). Atorvastatin treatment, 100 mg/kg daily *o.g.*, alone produced no significant effects on the tumor growth compared with that in the vehicle group (mean RTV, atorvastatin *vs.* vehicle: 14.03 *vs.* 14.58). However, when combined with atorvastatin, the anti-cancer activity of irinotecan was greatly extended, with a T/C value of 0.56 (mean RTV: irinotecan + atorvastatin *vs.* vehicle: 8.18 *vs.* 14.58, *p* = 0.0003; irinotecan + atorvastatin *vs.* irinotecan: 8.18 *vs.* 11.81, *p* = 0.036).

Furthermore, we performed immunofluorescence analysis of the xenografted tumors from the nude mice administrated by irinotecan and/or atorvastatin. As shown in revised Figure 6E, YAP was mainly located in the cytosol of the tumor cells obtained from atorvastatin- or the combination-administrated mice, suggesting that the nuclear translocation of YAP was suppressed by atorva and irinotecan+atova. However, irinotecan imposed minimal effect on the nuclear localization of YAP (Figure 6E), which was consistent with the results obtained from cellular models (Figure S4). In line with Figure 6E, the p-YAP levels in atorvastatin- and combination groups were enhanced, denoting the prohibition of nuclear translocation of YAP in these groups (Figure 6F).

Together with the results of our *in vitro* studies, these results suggested that the administration of statins might be a promising new strategy to alleviate or overcome the hypoxic resistance of HCC to SN38, probably through the inhibitory effects on hypoxic-activated YAP

## DISCUSSION

Because it has been shown that HIF-1α is the master regulator of diverse cellular processes under hypoxic conditions, a wide range of pharmacological approaches have been developed to interrupt its activity. Several small molecules were found to alleviate the hypoxic drug resistance and arrest the tumor xenograft growth through the inhibition of HIF-1α [4, 39]. Despite these efforts, an agent that targets HIF-1α is still not under clinical application. Therefore, there is an urgency to identify an alternative “druggable” molecule, that 1) mediates (or acts coordinately with HIF-1α) the malignant cellular behavior such as hypoxic resistance and 2) could be easily targeted with small molecules agents, thus opening the unexplored routes for therapeutic intervention aiming at combat with drug resistance under hypoxia.

Recent studies reported that YAP overexpression and activation is correlated with poor prognosis of cancer patients [18, 40], likely because it promotes tumor progression [27, 41] and/or metastasis [42–44]. Accumulating evidence suggests that the activated YAP contributes to the drug resistance of cancer cells. Using a genetic screen, Lin *et al.* identified that YAP plays a key role in promoting the drug resistance to RAF- and MEK-targeted cancer therapies [45]. Others found that YAP conferred resistance toward chemotherapeutic agents such as 5-FU [46], cisplatin [47, 48], taxol [48, 49], and bleomycin [48] in a variety of cancer models, including ovarian cancer [48], squamous carcinoma [47], and colon cancer [46]. However, although a recent report [30] demonstrated the activation of YAP under hypoxic conditions, the contribution of activated YAP to resistance to anticancer agents remained to be investigated. Given the important roles of YAP in promoting the anti-apoptotic and pro-survival mechanisms of cancer cells [50, 51], it appeared likely that the YAP pathway also played a key role in promoting the resistance to therapeutic agents. Here, for the first time, we established the link between the nuclear translocation of activated YAP and the hypoxic resistance toward SN38 in HCC models.

Consistent with the results of an earlier study using breast cancer cell models [30], we found that YAP was activated in HCC cells under hypoxia and in the hypoxic regions in xenografted tumor models. Our results showed predominant nuclear localization of YAP, prevented the cytoplasmic retention and subsequent degradation, thus increased the protein stability of YAP (Figure S3), and activated YAP target genes under hypoxic conditions (Figure 2). Intriguingly, the inhibition of YAP pathway, which prevented the nuclear translocation of YAP (Figure 5), either by YAP knockdown or using statins abolished the hypoxia-induced resistance of HCC cells toward SN38, as evidenced by the decreased cell survival and/or increased apoptosis rates of YAP knockdown cells treated with SN38, especially those under hypoxia (Figures 3 and 6). More importantly, the concurrent administration of SN38 and atorvastain in the HepG2 xenografted nude mice models produced improved anticancer effects (Figure 6).

Several small molecules have been shown to interfere with the activation of the YAP pathway. Specifically, statins, inhibitors of the mevalonate metabolic pathway widely used for the treatment of hypercholesterolemia, were shown to potently inhibit the YAP pathway by preventing the nuclear accumulation of YAP (statins) [37, 52] and thereby interfering with the transcription of YAP target genes. By doing so, statins were endowed with the ability to suppress cancer metastasis, which may partially explain the anti-cancer effects of these inhibitors. However, whether statins can improve the efficiency of chemotherapy under hypoxia remained unknown. The results on the sensitization of hypoxic cells by statins to SN38 treatment extended the current knowledge regarding the beneficial effects of these compounds by providing evidence for the feasibility of using statins to treat aggressive tumors that present hypoxic resistance to SN38 or other Topoisomerase I inhibitors. The alleviation of the hypoxic-resistance by statins may largely be due to its interference with the activation of YAP under hypoxic conditions.

In agreement with the results of an earlier study [30], we found that under hypoxia, the phosphorylation level of YAP decreased, which was accompanied by a significant increase in the nuclear localization of YAP and increased accumulation of total YAP protein (Figure 2). In mammalian cells, the subcellular localization of YAP is predominantly modulated by the LATS1/2 kinase cascade. Activated LATS1/2 induces the phosphorylation of YAP, leading to its ubiquitination and subsequent degradation by proteasome. When provoked by specific signals such as loss of cell-cell contact or disturbed cell polarity, the LATS1/2-mediated suppression of YAP activation is relieved, allowing the nuclear translocation of this transcriptional co-activator, where it binds to TEADs to activate the target gene transcription. In the present study, we found that the p-LATS1 level was reduced following the hypoxic stimuli, but this reduction in the p-LAST1 levels was attenuated in statin-treated hypoxic HCC cells (Figure 5). These results suggested that the HMGCR activated under hypoxia might contribute to the dephosphorylation of LATS1 by modulating RhoA [52]. In cells treated with statins, the total LATS1 and LATS2 protein level was also slightly decreased (Figure 5), which was consistent with the earlier report on the hypoxic degradation of LATS2 mediated by the accumulated SIAH2, an E3 ligase of LATS2. Given the high degree of homology and significant overlap in the function of LATS1 and 2, they are likely to have similar features, particularly on the domains modulated by SIAH2 or the other hypoxia-responsive E3 ligase(s).

Notably, HIF-1α, the master regulator of hypoxia, appeared not to contribute to the hypoxic activation of YAP. As shown in Figure 3, neither its knockdown nor its accumulation following the exogenous expression or CoCl_2_ treatment had an effect on the nuclear translocation. Although CoCl_2_ exposure reduce p-YAP level, the total YAP was not accumulated, implicating that some other post-translational modification of YAP, for instance, ubiquitination, caused by CoCl_2_ [34] may interfere with YAP stability (unpublished data). These findings suggested that the activation of YAP pathway under hypoxia was HIF-1α-independent. However, according to Ma's study, the nuclear YAP may aid in stabilizing the HIF-1α by binding and maintaining its activity [30]. Thus the present results underline the significance of YAP in the hypoxic microenvironment of solid tumors.

## MATERIALS AND METHODS

### Cell lines and cell culture

Human hepatocellular carcinoma cell lines HepG2, SMMC-7721 and Bel-7402 were purchased from Cell Bank of China Science Academy (Shanghai, China) and maintained in DMEM or RPMI-1640 medium (Gibco, Grand Island, New York, USA) plus 10% heat-inactivated fetal bovine serum and incubated at 37°C in a 5% CO_2_ atmosphere. Penicillin (100 U/ml) and streptomycin (100 U/ml) were added in the medium.

### Gene transfection and RNAi

Cells were seeded on 6-well plates, and the medium was replaced with Opti-MEM I Reduced Serum Media (Invitrogen) containing 20.0 nM siRNA (GenePharma, China) and oligofectamine (Invitrogen) according to manufacturer's recommendations. The sense sequence of the YAP siRNA#1, YAP siRNA#2 and HIF-1α siRNA were 5′-GACAUCUUCUGGUCAGAGATT-3′ [53], 5′-GGUGAUACUAUCAACCAAATT-3′ [54] and 5′-CUGAUGACCAGCAACUUGATT-3′ [55], respectively. The pCMV6-HIF-1α plasmid or empty vector were transfected into cells using the X-tremeGENE HP DNA Transfection Reagent (Roche, Penzberg, Germany) as recommended by the manufacturer.

### Immunofluorescene (IF) staining

HepG2 cells were collected and inoculating subcutaneously into nude mice. After the tumor size reached a mean group size of 300–500 mm^3^, the hypoxia probe Hypoxyprobe^™^-1 (Pimonidazole Hydrochloride, PIMO) (60 mg/kg) was injected intraperitoneally to indicate tumor hypoxic regions. One hour later, the tumors were removed and cut into frozen slices. For detection of the intratumor and subcellular localization of YAP, the cells grown in glass bottom dishes (Thermo Scientific Nunc Lab-Tek) as well as the frozen slices were fixed with 4% paraformaldehyde, and blocked with 5% BSA, and incubated with anti-YAP antibody (Cell Signaling Technology, Beverly, Mass) and FITC-conjugated secondary antibody (Invitrogen). The slides were than stained with DAPI and imaged with Leica DMI 400B fluorescence microscope.

### Immunobloting analysis

Immunobloting analysis of proteins in cell lysates and nuclear fractions was performed as previously described [56, 57]. Primary antibodies used were as follows: anti-YAP (#4912), anti-p-YAP (#4911), anti-Myc-Tag (#2278), anti-Mst (#3682), anti-p-Mst (#3681), anti-LATS (#3477) and anti-p-LATS (#8654) were purchased from Cell Signaling Technology (Beverly, MA); anti-HIF-1α (#610959) was purchased from BD; anti-β-Actin (#Sc-47778), anti-Lamin B (#Sc-6216), anti-BNIP3 (#Sc-56167), and anti-HMGCR (#27578) were purchased from Santa Cruz (Santa Cruz, CA).

### Quantitative real time RT-PCR (qRT-PCR) analysis

Total RNA was prepared using the EasyPure RNA kit (Transgen Biotech, Beijing, China). The “TransScript One-Step gDNA Removal and cDNA Synthesis SuperMix” kit (Transgen) was then used to purify the RNA and synthesise single-stranded cDNA, followed by SYBR^®^-Green real-time PCR (Qiagen, Valencia, CA, USA). The following primers were used: YAP, 5′-CGAGCTCATGCCTCTCCAGCTTCTCTGCAG-3′ (forward) and 5′-GCGGCCGCTATAACCATGTAAGAA AGC-3′(reverse) [58]; GAPDH, 5′-GTCATCCATGACA ACTTTGG-3′ (forward) and 5′-GAGCTTGACAAAGT GGTCGT-3′ (reverse).

### Cell proliferation and cell death assay

Cell proliferation was assessed by the SRB assay [59] and BrdU assay [31], and the cell death was monitored by the Trypan blue exclusion assay [32]. The cell proliferation inhibition rate for each well was calculated as (ODcontrol cells − ODtreated cells)/ODcontrol cells × 100%. By using trypan blue exclusion, cell death in the cultures were measured as previously described.

### Antitumor activity *in vivo*

Human HepG2 xenografts were established by subcutaneously inoculating 5 × 10^6^ cells into nude mice. When the tumors reached a mean group size of 100 mm^3^, the mice were randomized into control and treatment groups to receive one of the following daily treatments for 14 days: vehicle (phosphate-buffered saline (PBS), intragastric administration (i.g.)); irinotecan (2.5 mg/kg, intraperitoneal (i.p.)); atorvastatin (100 mg/kg, i.g.); combination therapy (2.5 mg/kg irinotecan plus 100 mg/kg atorvastatin). Tumor volume (V) was calculated as V= (length × width^2^)/2. The relative tumor volume (RTV) was calculated as RTV = Vn/V0 (Vn was the tumor volume on day n; V0 was the tumor volume on the first day of treatment). Therapeutic treatment effects were expressed in terms of T/C = mean RTV of the treated group/mean RTV of the control group.

### Statistical analysis

Student's *t*-tests were performed as indicated in the figures. Results were considered significant when *p* < 0.05 (*p < 0.05, **p < 0.01 and ***p < 0.001).

## CONCLUSION

In summary, for the first time, we demonstrated that the activation of YAP under hypoxia was strongly correlated with the resistance of HCC cells toward SN38, a Topoisomerase I inhibitor. Hypoxia inhibited the LATS1 phosphorylation, which triggered the nuclear translocation of YAP and the subsequent activation of its target genes, contributing to the decreased anti-cancer activities of SN38 in hypoxic HCC cells. The present results clearly suggest that the MVA-HMGCR pathway, but not HIF-1α, plays a critical role in the hypoxia-induced activation of YAP, particularly because the HMGCR inhibitor statins effectively abolished the nuclear translocation and accumulation of YAP protein under hypoxic conditions. The SN38, when combined with statins or YAP siRNA, exerted significantly improved anticancer activity, as revealed by the enhanced inhibitory effects on HCC cell proliferation, sensitization to apoptosis, and enhanced *in vivo* antitumor efficiency. Collectively, our study not only provides a rational basis for the combined use of statins and irinotecan as a promising novel regimen against HCC in patients presenting hypoxic resistance but also identifies the YAP signaling pathway to be a promising new target for the therapeutic intervention of drug resistance under hypoxic conditions.

## SUPPLEMENTARY MATERIALS FIGURES


