# Supplementary material for: Nuclear translocation and activation of YAP by hypoxia contributes to the chemoresistance of SN38 in hepatocellular carcinoma cells
Source: Oncotarget. 2016 Jan 12;7(6):6933–47. doi: 10.18632/oncotarget.6903 (PMC4872759; doi:10.18632/oncotarget.6903)
Supplement: Supplementary file 1 [file oncotarget-07-6933-s001.pdf]

Nuclear translocation and activation of YAP by hypoxia contributes to the chemoresistance of SN38 in hepatocellular carcinoma cells

Supplementary Materials

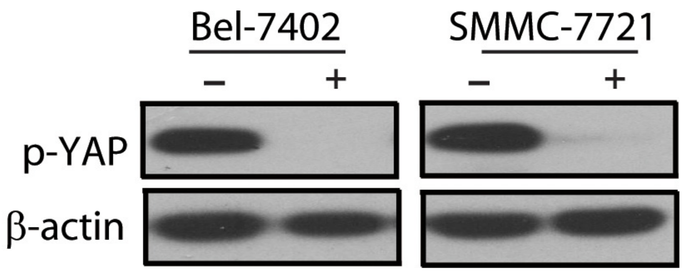

Supplementary Figure S1: The phosphorylated YAP was reduced under hypoxia in Bel-7402 and SMMC-7721 cells.

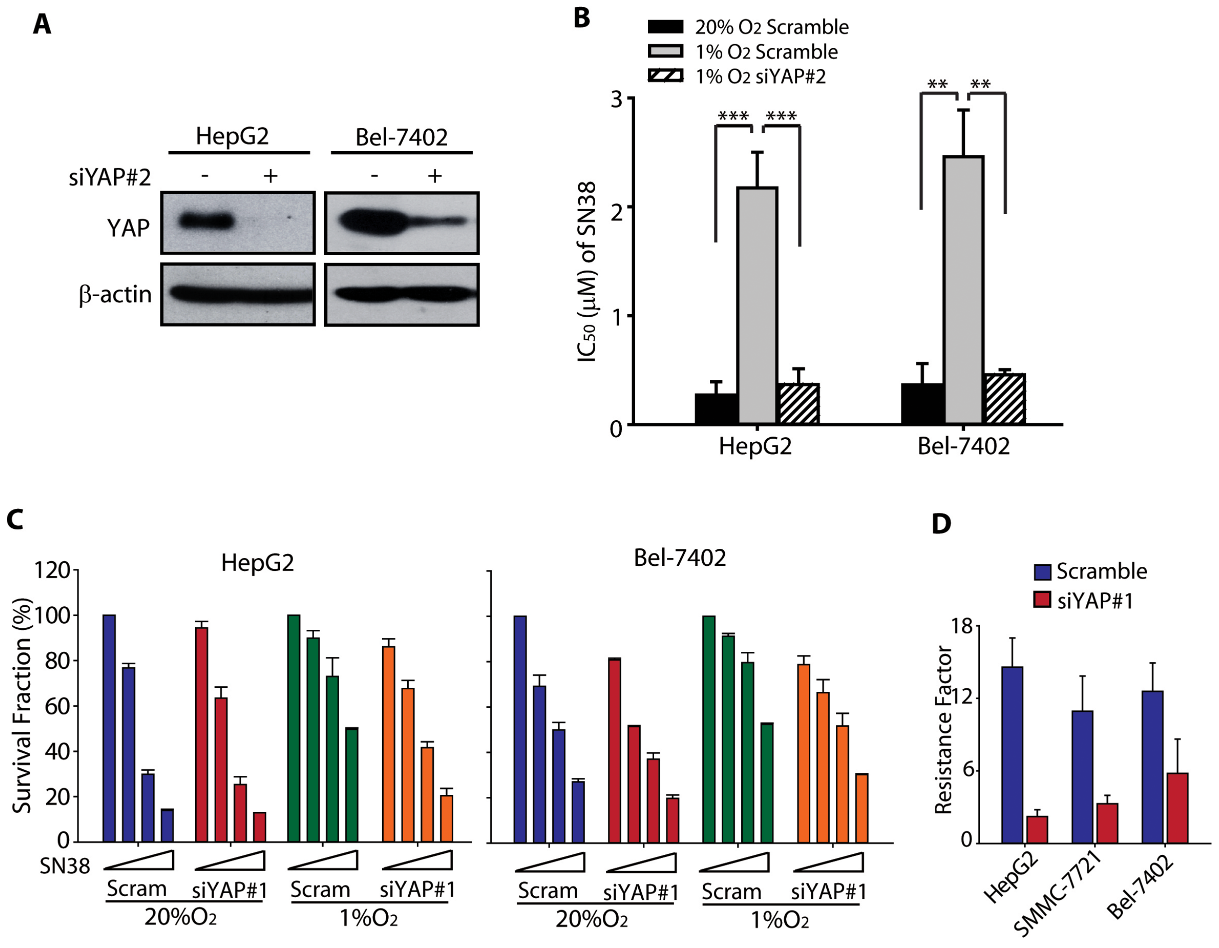

Supplementary Figure S2: (**A**) siYAP#2 sequence was introduced to silence YAP in HepG2 and Bel-7402 cells. (**B**) YAP depletion by siYAP#2 sensitized HepG2 and Bel-7402 cells to SN38 treatment under hypxoxia. (**C**) YAP silence preferentially increased anti-cancer activity of SN38 under hypoxia, compared to that under normoxia. (**D**) The hypoxic resistance was significantly attenuated by YAP knock-down.

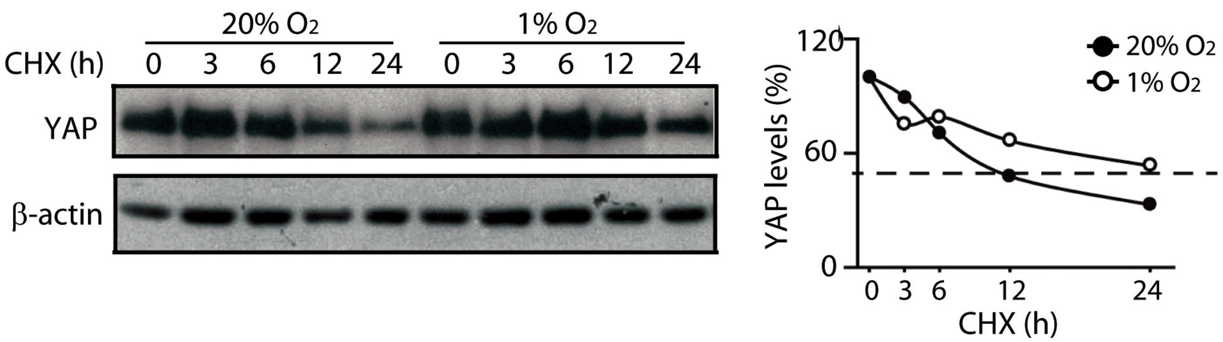

Supplementary Figure S3: The protein stability of YAP was enhanced under hypoxia.

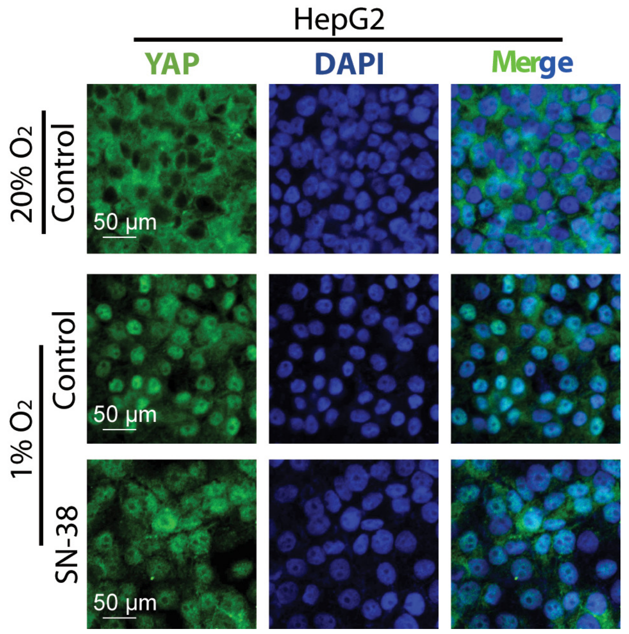

Supplementary Figure S4: SN38 imposed minimal effects on the nuclear accumulation of YAP under hypoxia.

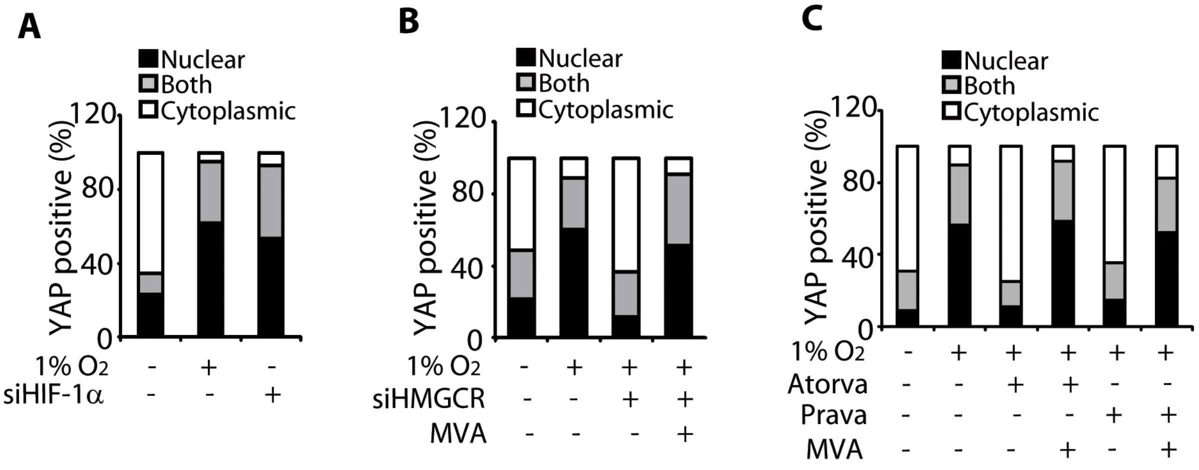

Supplementary Figure S5: The quantification of cellular distribution of YAP monitored by IF staining.
